# The Evolving Role of Gut Microbiota in the Management of Irritable Bowel Syndrome: An Overview of the Current Knowledge

**DOI:** 10.3390/jcm9030685

**Published:** 2020-03-04

**Authors:** Amir Mari, Fadi Abu Baker, Mahmud Mahamid, Wisam Sbeit, Tawfik Khoury

**Affiliations:** 1 Gastroenterology and Endoscopy Units, The Nazareth Hospital, EMMS, Nazareth, Faculty of Medicine in the Galilee, Bar-Ilan University, Safed 1311502, Israel; mahmudmahamid@yahoo.com (M.M.); tawfikkhoury1@hotmail.com (T.K.); 2 Gastroenterology Department, Hillel Yaffe Medical Center, Hadera 38100, Israel; fa_fd@hotmail.com; 3 Gastroenterology Department, Sharee Zedek Medical Center, Jerusalem 9103102, Israel; 4 Gastroenterology Department, Galilee Medical Center, Nahariya, Israel, Faculty of Medicine in the Galilee, Bar-Ilan University, Safed 1311502, Israel; wisams@gmc.gov.il

**Keywords:** gut, microbiome, IBS

## Abstract

The intestinal microbiota is one of the most rapidly evolving areas in biology and medicine. Extensive research in the last decade has escalated our understanding of the role of the microbiota in the pathogenesis of several intestinal and extra-intestinal disorders. Marked by high prevalence, substantial morbidity, and enormous costs, irritable bowel syndrome (IBS) is an important chronic gastrointestinal disorder that is widely encountered by gastroenterologists. Despite advances in our understanding of its pathophysiology, curative interventions have yet to be discovered, and therapeutic approaches remain symptom-driven. Recently, accumulating evidence has enlightened the possible impact of an imbalanced gut microbiome in the pathogenesis of IBS. In fact, several studies have documented altered microbiota in patients, while others have shown that IBS severity was associated with a distinct microbiota signature. These findings may pave the way for the use of microbiota manipulation strategies as an attractive option for IBS management, and may have an essential role in efforts to reduce the societal and economic effects of this ever-growing disorder. In this review, we have outlined the results of the latest research on the association between microbiota and IBS and their implications for the clinical management of affected patients.

## 1. Introduction

**Figure 1:**
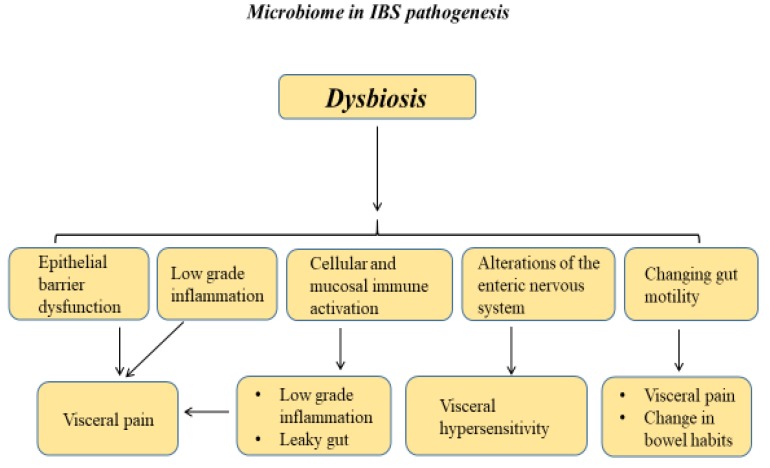
Role of the microbiome in irritable bowel syndrome (IBS).

Irritable bowel syndrome (IBS) is a common functional gastrointestinal disorder characterized by chronic recurrent abdominal pain, changes in bowel habits, and other symptoms such as bloating and flatulence. Based on the Rome IV criteria, four subtypes of IBS exist depending on the predominant stool pattern, including IBS with constipation (IBS-C), IBS with diarrhea (IBS-D), IBS with mixed bowel habits (IBS-M), and unclassified IBS [1,2]. IBS has a global prevalence of approximately 11% and is associated with several comorbidities, such as anxiety, depression, fibromyalgia, migraines, chronic pelvic pain, and others [3,4]. IBS is a major socioeconomic burden because affected patients utilize more healthcare resources with reduced work productivity when compared to the healthy population [5]. IBS is a complex heterogeneous condition with a multifactorial pathogenesis. Proposed mechanisms involved in the pathogenesis of IBS include visceral hypersensitivity, gut–brain axis alterations, disorders in the epithelial barrier integrity leading to abnormal mucosal intestinal permeability, changed intestinal motility, immune system activation, food intolerance, low-grade inflammation, altered enteroendocrine pathways signaling, genetic basis (e.g., mutation in the SC5NA gene encoding a sodium channel ion; a number of single-nucleotide polymorphism studies have also identified polymorphisms in genes associated with IBS pathogenesis including genes coding for serotonin signaling, immune regulation, and epithelial barrier function), and the evolving concept of dysbiosis in the gut microbiota (Figure 1) [6,7,8,9,10,11,12,13,14,15].

Gastrointestinal (GI) microbiota are the most relevant microbial community in the body and belong to the so-called microbiota which includes all microorganisms living in the human body. The intestinal microbiota includes bacteria, archaea, fungi, eukaryotes, and viruses. Most of the bacteria in the GI tract are represented in four main bacterial phyla: Firmicutes, Bacteroidetes, Proteobacteria, and Actinobacteria [16,17]. Trillions of microorganisms reside in the GI tract, with the highest density in the colon. However, most of them remain uncharacterized. Since this number is approximately equal to 10 times the total number of body cells, the interest in microbiota study for intestinal and extra-intestinal diseases is not surprising [18,19,20,21,22]. Advances in molecular biology techniques have significantly contributed to microbiome research. The most common technique to analyze the composition of the gut bacteria is marker gene sequencing, generally using the 16S rRNA gene [23,24,25]. Advantages of this method are related to its simplicity and low cost.

The aim of this review is to overview the most up-to-date literature about the evolving role of gut microbiome manipulation in IBS management, with a focus on probiotics, prebiotics, antibiotics, and fecal transplantation.

## 2. Methods

A search for studies published before January 2020 was performed in the PubMed and Embase databases. All authors participated in the search process. We looked for the terms “irritable bowel syndrome”, “microbiota”, “treatment”, “prebiotic”, “probiotic”, “synbiotic”, “FODMAP”, “meta-analysis”, “randomized”, “clinical”, “bifidobacteria”, “lactobacillus”, “firmicutes”, “bacteroidetes”, “methane”, “methanogen”, “diet”, “fecal transplantation”, “bacteriophage”, and “fungi”, mainly focusing on the literature that describes effects on microbiota, clinical studies, and therapeutic effects in IBS. The search was restricted to articles in the English language.

## 3. The Microbiome in IBS

A rash of research activity in this field from the last decade has led to the evidence that a disruption in the biodiversity, richness, and composition of the gut microbiota—a process named dysbiosis—plays a key function in the pathogenesis of IBS [26]. Dysbiosis can take place through several mechanisms: the overgrowth or vanishing of specific bacteria species, alterations in the relative richness of bacteria, and lastly, by mutation or gene transfer [27].

In IBS patients, GI dysbiosis has been associated with a visceral increased perception of pain and enhanced mucosal permeability that is provoked by the defective mucosal epithelial barrier, interfering with gut immune homeostasis and subsequently promoting gut inflammation and enhancing cellular and mucosal immune activation [28,29]. Moreover, it has also been associated with changing gut motility, low-grade chronic inflammation, alterations of the enteric nervous system, and vagal afferents neurons as well as brain functions [30,31,32]. In opposition, gut microbiota can be affected by the brain activity on intestinal motility, secretion, and immune function, generating the microbiota–gut–brain axis [12,33].

Microbiological and infectious bases of IBS pathogenesis have been previously established by several groups. Halvorson et al. reported a seven-fold increased risk of post-infectious IBS after acute infectious gastroenteritis [34]. Moreover, therapeutic interventions that manipulate the gut microbiota such as antibiotics, prebiotics, probiotics, and fecal microbial transplantation have been linked to improvements in IBS symptoms [35,36,37]. Several studies aiming to characterize and map the microbiome signature of IBS have shown divergent results. Nonetheless, data suggest that there is a relative richness of proinflammatory bacterial species containing Enterobacteriaceae, with a parallel decline in *Lactobacillus* and *Bifidobacterium* [38,39]. A decreased percentage of *Lactobacillus* and *Bifidobacterium* species has also been described in the IBS microbiota, leading to disturbances in short-chain fatty acid production and in immunologic and bactericidal activity, with a negative effect on microbiota function and stability [40,41,42,43,44,45,46]. Interestingly, the Firmicutes/Bacteroidetes ratio is a possible indicator of bacterial population shifts, and both high and low ratios of Firmicutes/Bacteroidetes have been reported in IBS patients [47,48,49,50]. These contrasts may be explained by differences in technical methods and subtypes of IBS, as well as the severity of IBS [38]. Several groups examined gut microbiota in different subtypes of IBS and compared the microbiota texture between different IBS subtypes as well as different IBS symptoms [48,51,52]. A study by Ringel-Kulka et al. using fecal samples from 60 patients with IBS and 20 healthy controls revealed major variances in the microbiota between the different subtypes of IBS based on clinical symptoms of abdominal bloating and bowel habits (IBS-D, IBS-C, or IBS-M type) [53]. A study examining both fecal and colonic mucosal microbiota in patients with chronic constipation found that their mucosal microbiota differed from those of the controls, with a higher abundance of Bacteroidetes species in the patients than in the controls. However, although the profile of the colonic mucosal microbiota discriminated between these two cohorts with a high level of accuracy, this finding was independent of colonic transit time. In contrast, the profile of the fecal microbiota was associated with colonic transit, but not with the clinical diagnosis of constipation [54]. Putting all of this together, numerous evidence is emerging regarding the link between microbiota and IBS pathogenesis, making microbiota manipulation strategies an attractive option for IBS management.

## 4. Therapeutic Interventions for Microbiome Manipulation in IBS

### 4.1. Probiotics

Historically, the theory of probiotics was described by Elie Metchnikoff in 1908, who observed that fermented foods—principally those fermented by lactic acid bacteria—had favorable effects on human wellbeing and longevity. According to the most updated definition based on the Food and Agriculture Organization as well as the World Health Organization, probiotics are defined as “live microorganisms that, when administered in adequate amounts, confer a health benefit on the host” [55]. Probiotics related to IBS pathogenesis are mainly those containing *Lactobacillus* and *Bifidobacterium* species [56]. Tentatively, probiotics may promote a favorable modulation of altered gut microbiota by several mechanisms: reducing the number of competing pathogens by both the production of antimicrobial substances and interfering in intestinal mucosal adhesion [57,58], modulating the metabolism of biliary salts [59], reducing low-grade inflammation [60], and regulating immune activation as well as gut motility [61]. Several meta-analyses of randomized controlled trials (RCTs) that assessed the effects of single probiotic strains, compared to a placebo in relieving IBS related symptoms [35,59,62], have concluded that probiotics are more efficient when compared to placebos in relieving global IBS symptoms including bloating abdominal pain. In safety terms, all studies have reported comparable rates of adverse events to the placebo arms. In an Iranian IBS cohort, Jafari and colleagues observed the effects of combinations of strains of *Bifidobacterium*, *Lactobacillus*, and *Streptococcus* genera [63]. The main finding was that 85% of patients in the probiotic group reported satisfactory relief of general symptoms compared with 47% in the control group (*p* < 0.01). In general terms, probiotics seem to have favorable effects on improving IBS symptoms, with an excellent safety profile. Nonetheless, more randomized controlled trials are warranted to better define some concerns such as treatment duration and optimal strain, and to better study personalized treatment.

### 4.2. Prebiotics

Since 2007, prebiotics have been defined as a “nonviable food component that confers a health benefit on the host associated with modulation of the microbiota” [64]. Prebiotics are basically classified as disaccharides or oligosaccharides and are resistant to enzymatic and chemical breakdown until they reach the colon, where they are fermented by colonic bacteria, stimulating the generation of microbial metabolic products such as short-chain fatty acids (acetate, butyrate, and propionate) [65]. Short-chain fatty acids give several benefits to the colonocytes, such as an energy source, regulation of electrolytes and water absorption, enhanced blood flow, and oxygenation [66]. Moreover, probiotics may promote host health and modulation of GI motility, reduction in visceral hypersensitivity, downregulation of low-grade mucosal immune activation, improvement of epithelial permeability, enhancement of gut–brain communication, and restoration of intestinal dysbiosis. Thus, these data provide a mechanistic rationale for a role of prebiotics in managing IBS symptoms. Indeed, several clinical studies have examined prebiotics’ performance in ameliorating symptoms of functional bowel disorders. A handful of RCTs evaluating the efficacy and safety of prebiotics in IBS [67,68] have been performed. In 1999, a small, double-blind crossover trial of oligofructose published by Hunter and colleagues [69] showed no therapeutic value in IBS patients. One year later, a randomized double-blind trial on almost 100 patients with IBS receiving either fructo-oligosaccharide or placebo for 12 weeks reported no statistically significant improvement in symptoms [70]. The first study to report a beneficial effect was a cross-over, single-blinded trial including 60 Rome II-defined IBS patients. Patients were randomized for four weeks to receive a low or high dose of trans-galacto-oligosaccharide or placebo. Both the low and high-dose arms experienced a significant improvement in stool consistency (*p* < 0.05), flatulence (*p* < 0.05), and bloating (*p* < 0.05) as well as a reduction in mean subjective global assessment (The subjective global assessment of relief was recorded at weekly intervals during the course of the study scored from 1–5. 1 = completely relieved, 2 = considerably relieved, 3 = somewhat relieved, 4 = unchanged, and 5 = worse) [71].

Interestingly, the prebiotic but not the placebo significantly enhanced fecal bifidobacteria [71]. A 12-week administration of partially hydrolyzed guar gum in a randomized, double-blind, placebo-controlled study led to a significant improvement in bloating and gasses scores with no effect on other reported IBS symptoms or quality of life scores, leading the authors to support its administration for IBS patients with an expected clinical effect on bloating and gasses [72]. In safety terms, all studies reported comparable rates of adverse events to the placebo arms. In conclusion, prebiotic use in IBS patients have yielded mixed to positive results, but further studies to address the combination, duration, and different aspects of effects on IBS are still warranted.

### 4.3. Non-Absorbable Antibiotics

Non-absorbable antibiotics, mainly rifaximin, have been shown to be safe and effective for the treatment of IBS with the diarrhea predominant type. Rifaximin, a rifamycin derivative, is a broad-spectrum, non-absorbable antibiotic which targets aerobic and anaerobic bacteria residing in the GI tract. Less than 1% of rifaximin is absorbed in the systemic circulation, making it very safe with extremely low toxicity and adverse events rates [73]. The proposed mechanisms of action of the non-absorbable antibiotics are the reduction of the amount of inhabitant GI bacteria, changes in bacterial structure, reduction of low-grade inflammation, and amelioration in gut permeability [74]. Rifaximin is the best-studied non-absorbable antibiotic for symptoms relief in IBS. The TARGET 1 and TARGET 2 trials, both designed as double-blinded, placebo-controlled, multi-center studies, have shown good efficacy of rifaximin for IBS symptoms relief [75]. In TARGET 1 and TARGET 2, a total number of 1258 patients with mild to moderate symptoms of IBS were randomized either to receive rifaximin 550 mg three times a day for 14 days or a placebo. Relief of IBS symptoms, after one month from the end of treatment, was reported more significantly among patients in the rifaximin group compared with those in the placebo group (40.7% vs. 31.7%, *p* < 0.001), with a comparable adverse events occurrence in both groups [75]. TARGET 3, a randomized, placebo-controlled study including 2579 patients with IBS, revealed that the durability of symptoms relief among patients with IBS-D responding to a 14-day course of rifaximin was reduced by 50% after 70 days from the end of treatment [76]. With a second treatment course, the most significant benefit was the relief of stool urgency and abdominal bloating [76].

### 4.4. Fecal Microbiota Transplantation

**Figure 2:**
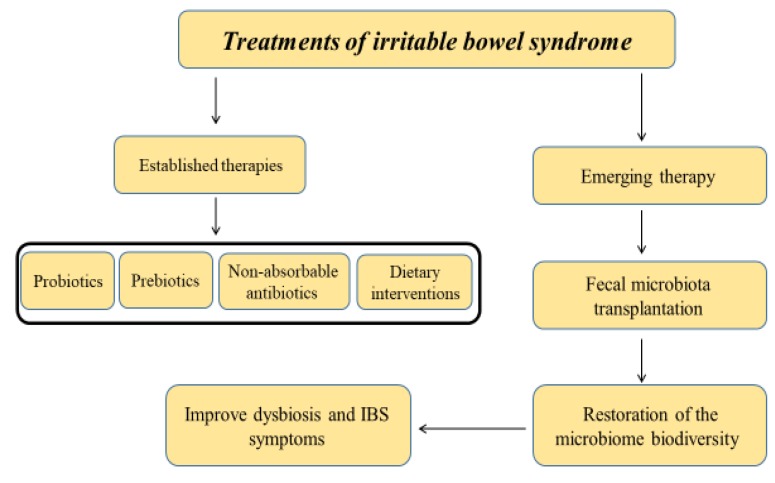
Treatments of irritable bowel syndrome (IBS) by targeting the gut microbiome.

Fecal microbiota transplant (FMT), also known as a stool transplant, is the process of transplantation of fecal bacteria from a healthy individual into a recipient. FMT involves the restoration of the colonic microflora by introducing healthy bacterial flora through the infusion of stool (e.g., via colonoscopy, enema, rogastric tubeor by mouth in the form of a capsule containing freeze-dried material) obtained from a healthy donor. To date, FMT has been approved for the treatment of resistant *Clostridium difficile* infections [77,78], and in this context, it has been shown to be an effective therapy [79]. However, its therapeutic effect among IBS patients is still emerging. As reported above, in the last few years, several studies have underlined the role of dysbiosis among patients with IBS, with lower *Lactobacillus* spp. in IBS-D and increased loads of *Veillonella* spp., and genera *Coprococcus*, *Collinsella,* and *Coprobacillus* [42,80]. Other data revealed a decrease in the biodiversity of microbiota in the fecal composition of IBS-D patients [51]. Therefore, targeting the gut microbiota composition might be a promising therapy in IBS. However, among IBS patients, FMT has shown conflicting results. A previous randomized controlled study showed beneficial effects of FMT on IBS symptoms. In this study, 55 patients with IBS-D or IBS-M received 50–80 g of feces mixed with 200 mL of isotonic saline and 50 mL of 85% glycerol, administered to the cecum by colonoscopy, and were compared to 28 patients who received a placebo. Patients treated with FMT showed a significant clinical response at three months compared to those in the placebo group (65% vs. 43%, *p* = 0.049) [39]. These findings were further confirmed by other recent studies showing a positive effect on IBS symptoms after transplant, as 70–85% and 45–60% of patients reported symptomatic relief in the first three months and six months after FMT, respectively [81,82]. On the contrary, a recent study including 52 IBS adult patients who were randomized to either active FMT or placebo capsules administered for 12 months did not show beneficial effects favoring FMT at three months. In fact, a significant improvement in IBS symptom scores was observed at three months favoring the placebo (*p* = 0.012), and after three months, the results obtained by an IBS quality of life questionnaire were in favor of the placebo (*p* = 0.003) [83]. Moreover, a recent meta-analysis including eight single-arm trials (SATs) and 5 RCTs did not report beneficial effects of FMT among patients with IBS (relative risk = 0.93, 95%, confidence interval (CI (confidence interval) 0.50–1.75, *p* = 0.83 for RCT), while in the SAT 59.5% of patients (95% CI 49.1–69.3) showed a significant improvement [84]. Given the controversies regarding the published data, FMT-based treatments for IBS are still not widely accepted among gastroenterologists, as they are concerned about effectiveness and safety profile [85]. Therefore, the role of FMT must be further addressed by randomized, double-blind, placebo-controlled studies. Recent international consensus of stool banking for FMT have involved experts from Europe, North America, and Australia who proposed consensus guideline statements regarding several issues in stool banking, including the selection of donors and screening objectives, collection and processing of stool samples, monitoring of outcomes, ethical issues, and the evolving role of FMT in *Clostridioides difficile* infection and other diseases encountered in daily clinical practice [86]. Figure 2 demonstrates the established and evolving therapeutic options for IBS.

### 4.5. Dietary Intervention

Dietary modifications constitute one of the first choices of treatment for IBS patients [87]. Indeed, a careful history may reveal patterns of symptoms linked to specific food consumption. Although debatable, a high-fiber diet has traditionally been encouraged particularly in IBS-C patients, given the absence of serious side effects and its potential benefit [88,89]. In recent years, there has been a growing clinical and scientific interest in the use of a diet low in FODMAPs (fermentable oligosaccharides, disaccharides, monosaccharides, and polyols) in IBS patients. Since its introduction, reports in the literature including several RCTs have reported the efficacy of a low-FODMAP diet in improving global IBS symptoms, visceral pain, bloating, and quality of life [90,91,92,93,94,95,96]. However, large and long-term RCTs are still lacking, and various concerns have been raised including diet complexity and cost, risk of nutritional deficiencies, and importantly, an unclear impact on gut microbiome [97,98,99].

A focus on the role of a gluten-free diet (GFD) in IBS has grown recently, with studies demonstrating the induction of symptoms following gluten consumption in IBS patients [100]. However, evidence to support gluten avoidance in IBS has been conflicting. Moreover, a recent report has suggested that fructans rather than gluten protein are responsible for the symptomatic improvement reported in a gluten-free diet [101]. Further studies are required to evaluate the effect of a GFD on nutritional status, gut microbiota, and long-term outcomes.

## 5. Conclusions

In conclusion, in the current review we have provided a focused summary of the latest literature on the potential role of gut dysbiosis in the pathogenesis of IBS and discussed the translation of microbiota-modifying strategies as a novel therapeutic option for this disorder (Figure 1). There is strong growing evidence supporting microbiome-based therapeutic approaches with dietary intervention, probiotics, prebiotics, non-absorbable antibiotics, and FMTs for the treatment of IBS (Table 1). Nonetheless, more knowledge is needed to better address the role of microbiome-based therapeutic interventions in the clinical management of IBS. Several future microbiome-based therapeutic options are being explored and investigated, including genetic engineering of bacteria, personalized microbiota manipulation, postbiotics, and bacteriophage therapy.

## Figures and Tables

**Table 1:** Summary of the meta-analysis studies reporting efficacy of therapeutic interventions in irritable bowel syndrome (IBS) based on overall global IBS symptoms.

**Meta-Analysis of RCT Studies of Probiotics vs. Placebo**					
**Reference**	**Number**		**Primary Endpoint as Dichotomous Variable (RR ± 95% CI)**		***p*-Value**
	**Studies**	**Patients**	**Clinical Improvement**	**Symptoms Persistence**	
Didari T., 2015 [59]	15	1793	2.43 ± 1.13–5.21	-	0.02
Zhang Y., 2016 [35]	21	1639	1.82 ± 1.27–2.6	-	<0.001
Liang D., 2019 [102]	14	1695	1.27 ± 1.13–1.44	-	<0.001
McFarland L.V., 2008 [103]	23	1404	0.77 ^pooled^ ± 0.62–0.94	-	<0.001
Connell M., 2018 [104]	5	243	1.39 ± 0.99–1.98	-	0.06
Ford A.C., 2014 [63]	23	2575	-	0.79 ± 0.7–0.89	<0.0001
Tiequn B., 2015 [105]	6	273 167	17.62 ^pooled^ ± 5.12–60.65 for adults 3.71 ^pooled^ ± 1.05–13.11 for children	- -	<0.00001 0.04
Ritchie M.L., 2012 [106]	16	-	0.77 ± 0.65–0.92	-	-
Horvath A., 2011 [107]	3	167	1.7 ± 1.27–2.27	-	0.0004
Hoveyda N., 2009 [108]	7	425	1.6 ± 1.2–2.2	-	0.0007
Moayyedi P., 2010 [109]	10	918	-	0.71 ± 0.57–0.88	0.002
Nikfar S., 2008 [110]	8	1011	1.22 ± 1.07–1.4	-	0.004
McFarland L.V., 2008 [103]	20	1404	0.77 ^pooled^ ± 0.62–0.94	-	<0.001
**Meta-Analysis of RCT Studies of Prebiotics vs. Placebo**					
Wilson B., 2019 [67]	11	729	OR 0.62 ± 0.07–5.69	-	0.67
Ford A.C., 2018 [68]	Trials for prebiotics were sparse and no definite conclusions could be drawn				
Ford A.C., 2014 [63]					
**Meta-Analysis of RCT Studies of Fecal Microbiota Transplantation vs. Placebo**					
Myneedu K., 2019 [84]	5	262	0.93 ± 0.5–1.75	-	0.83
Xu D., 2019 [111]	4	254	0.93 ± 0.48–1.79	-	0.83
Laniro G., 2019 [112]	5	267	-	0.98 ± 0.58–1.66	0.94
**Meta-Analysis of RCT Studies of Non-Absorbable Antibiotics Rifaximin vs. Placebo**					
Ford A.C., 2018 [68]	5	3610	-	0.84 ± 0.79–0.9	0.0002
Li J., 2016 [113]	4	1803	1.19 ± 1.08–1.32	-	0.0008
Menees S.B., 2012 [75]	5	1803	1.57 ± 1.22–2.01	-	<0.001

RCT: Randomized Controlled Trials; CI: Confidence Interval; ^pooled^: Pooled RR.
